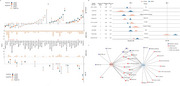# Roles of *APOE* e4 in Modulating the Relationship of Non‐genetic Risk Factors with Dementia and Alzheimer’s disease: A System Review and Meta‐analysis of 170 Longitudinal Studies

**DOI:** 10.1002/alz70861_108125

**Published:** 2025-12-23

**Authors:** Wei Xu, Chen‐Chen Tan, Liangyu Huang

**Affiliations:** ^1^ Qingdao Municipal Hospital, Qingdao University, Qingdao, Shandong China; ^2^ Qingdao Municipal Hospital, Qingdao, 266071 China

## Abstract

**Background:**

The interplays between risk factors and apolipoprotein E4 allele (*APOE* e4) could play crucial roles in influencing dementia occurrence. However, the observational evidence remains fragmented. This study aims to comprehensively evaluate whether the risk factor profile of dementia varied by the presence of *APOE* e4.

**Method:**

A systematic search of PubMed, EMBASE, and the Cochrane Library was conducted up to June 2023. Population‐based longitudinal studies were included if they reported associations of modifiable or nonmodifiable risk factors with all‐cause dementia (ACD) or Alzheimer's disease (AD) stratified by *APOE* e4 status. The multivariable‐adjusted effects were separately combined using random‐effects models in *APOE* e4 carriers and non‐carriers. Meta‐regression analyses were performed to test stratification effects by *APOE* e4. The study protocol was pre‐registered in PROSPERO and the registration number is CRD42024612115.

**Result:**

A total of 170 eligible literatures with 173 factors were identified, of which 112 with 48 factors were included in the meta‐analysis, comprising 1,202,988 *APOE* e4 carriers and 2,190,941 non‐carriers. Meta‐regression revealed stratification effects by *APOE* e4 for nine ACD risk factors. Among these, four factors (nonsteroidal anti‐inflammatory drugs, statins, frequent drinking, and high systolic blood pressure) showed significant associations only in *APOE* ε4 carriers, while five factors (light‐to‐moderate alcohol consumption, female, physical activity, diabetes mellitus, and loneliness) were significant only in non‐carriers. Moreover, the associations for six risk factors (aging, education, hypertension, cardiovascular disease, stroke, and depression) were not modulated by the presence of *APOE* e4 status. As for AD, meta‐regression analyses identified diabetes as a specific risk factor in *APOE* e4 non‐carriers and depression was found a risk factor independent of *APOE* e4. Subgroup analyses revealed four factors uniquely in *APOE* e4 carriers (NSAIDs use, vitamin E intake, high SBP, and loneliness) and other six factors specific to non‐carriers (aging, female, Mediterranean diet, physical activity, cardiovascular disease, and serum testosterone).

**Conclusion:**

*APOE* e4 could interact with other risk factors to influence dementia occurrence. Future studies are needed to validate these interactions and elucidate the underlying mechanisms, enabling precise prediction and prevention of dementia and AD.